# The recognition problem in collective optimal experience: the identity of generation and recognition as a structural event

**DOI:** 10.3389/fpsyg.2026.1861167

**Published:** 2026-07-01

**Authors:** Isabella Langwei, Bin Li

**Affiliations:** 1 Research Institute of Fujian-Taiwan Cultural Tourism and Health Industry, Longyan University, Longyan, Fujian, China; 2 Centre for Healthy Brain Ageing (CHeBA), UNSW Medicine & Health, University of New South Wales, Sydney, NSW, Australia

**Keywords:** bisosilin, body grammar, Bunun, collective optimal experience, critical convergence, pasibutbut, recognition criterion, structural event

## Abstract

The recognition of collective optimal experience remains structurally unresolved in psychology. Existing frameworks—from group flow to collective effervescence—have extended optimal experience to the collective level, yet they have generally treated individual reports as the primary endpoint for determining whether such experience has occurred, leaving unresolved how collective recognition might be established at the relational rather than individual level. This recognition problem is addressed through an empirical analysis of *bisosilin*, the evaluative term used by Bunun singers to determine whether their ritual polyphonic chant *pasibutbut* had reached completion. Drawing on two-stage qualitative fieldwork with 24 participants, the findings indicate that recognition does not depend on any individual’s internal state, but rather on whether a specific relational configuration—comprising sonic alignment, bodily coordination, and shared intentional orientation—has been achieved. On this basis, the study advances a central theoretical claim: collective optimal experience is not first generated and only subsequently recognized, but is established as a structural event in which generation and recognition are constituted as structurally inseparable aspects. To account for this process, two analytical frameworks are proposed. First, a three-layer model of body grammar (action, perceptual, and consciousness grammar) specifies the necessary structural conditions of collective optimal experience while showing that these conditions enable but do not establish it. Second, the process–uncertainty–alignment (PUA) model explains how these conditions are dynamically activated, leading to a nonlinear transition—termed critical convergence—at which point establishment occurs. This implies that collective optimal experience is not an internal state to be measured but a condition that becomes identifiable only insofar as it is collectively recognizable. Accordingly, retrospective self-reports capture the aftermath of establishment rather than its occurrence. This study does not introduce a new type of collective peak experience but instead redefines the generative mechanism and establishment condition of collective optimal experience as a structural event.

## 1 Introduction

Psychology has studied collective experience for decades; however, it has rarely addressed one question explicitly: by what criterion, and where, is a collective optimal state recognized as having occurred? This omission is not incidental. Across group flow, collective effervescence, and coordination-based accounts, the occurrence of collective experience has often been assessed through individual reports, which remain indispensable for many research purposes but do not by themselves specify how collective occurrence is recognized at the relational level. The present study therefore treats the problem not as a defect of self-report methodology, but as a question concerning the analytical unit through which collective occurrence is recognized. This suggests that the individual is not merely a methodological convenience, but the assumed locus of epistemic authority in determining whether optimal experience has occurred.

The individual-centered assumption has shaped much of optimal experience research and reflects a broader pattern that cultural psychology has long identified, in which psychological constructs developed in predominantly Western research contexts often treat the bounded individual as the default unit of analysis (Markus and Kitayama, 1991). Maslow’s peak experience (Maslow, 1964, 1971) and Csikszentmihalyi’s flow theory (Csikszentmihalyi, 1975, 1990) establish concentrated attention, perceived control, intrinsic motivation, and subjective enjoyment as the defining markers of heightened psychological states—all of which were identified through internal indicators and validated by individual reports. Even as the field expanded toward collective phenomena, this epistemic structure was preserved. Research on group flow, team flow, and shared flow demonstrates that optimal states can be socially scaffolded through shared goals, synchronized interactions, and mutual trust (Sawyer, 2003; Pels et al., 2018; Van den Hout and Davis, 2023; Hobson et al., 2025). Studies on collective effervescence and perceived emotional synchrony have extended the analysis to rituals, demonstrations, and communal events, thereby identifying shared uplift, identity fusion, and self-transcendence as collective phenomena (Páez et al., 2015; Wlodarczyk et al., 2020; Pizarro et al., 2022). However, in all of these frameworks, the occurrence of a collective state is confirmed by asking individuals how intensely they experienced it. Even in group flow research, collective experience is still confirmed through aggregated individual reports. The “we” of the collective is derived from the aggregation of private “I”s, and the recognition criterion remains individual-based.

Relational and enactive traditions have gone furthest in challenging this assumption. De Jaegher and Di Paolo (2007) demonstrate that social interaction can constitute a form of sense-making with genuine autonomy—an organized dynamic irreducible to the sum of individual mental states. Fuchs and De Jaegher (2009) extend this through mutual incorporation, showing how bodies form shared sensorimotor fields during interactions. These represent important developments in relocating experience to interaction. These approaches successfully relocate experience to interaction but leave unresolved the criterion by which such interaction is recognized as having achieved an optimal state. They address generation—the process by which collective experience comes into being—but not recognition—the moment at which it is identifiable as complete. This distinction is not a minor omission. It is the recognition problem itself. Without such a criterion, the distinction between an ongoing interaction and an established optimal state remains theoretically indeterminate.

These approaches provide essential insights into the dynamics of collective interaction. However, they leave open a distinct question concerning the recognition of completion. Sawyer's (2003) account of group creativity approaches a relational explanation, emphasizing emergent collective outcomes irreducible to individual contribution. However, even in Sawyer’s framework, the question of how the group itself determines that a given moment has succeeded—that the emergent product has reached completion—remains unaddressed. In other words, the establishment problem is not engaged. Coordination dynamics offer another entry point. Riley et al. (2011) have shown how actors in sustained interaction couple their degrees of freedom to form higher-order control structures, while Tognoli et al. (2020) describe stable coordinated states and self-organization in social interaction. These models explain how coordination emerges but not when it becomes recognizable as complete. Alignment occurs—but when does it become established? This question is outside the scope of these models. The joint action and error-correction literature faces an analogous boundary. Repp and Keller (2008) and Vesper et al. (2010) document with precision how timing adjustments and phase corrections maintain collective coordination. Accordingly, these studies describe how coordination is maintained, but they do not identify how that coordination is deemed as successful. Maintenance is not an establishment. The moment at which coordination crosses the threshold from occurring to being recognized as complete lies beyond what these frameworks address.

The convergence of these limitations points to a single conclusion: existing research explains how collective experience emerges but not how it is recognized as having occurred. The problem is not what collective experience is, but how it is recognized as having occurred—by what criterion and the location where that recognition occurs. This implies that the problem cannot be resolved by refining measurement instruments alone, because it concerns not how recognition is measured but where it is epistemically located. This issue is therefore conceptual rather than methodological.

Cross-cultural scholarship suggests that this location is not fixed. Han's (2015) analysis of *Shinmyeong* in Korean contexts, grounded in the cultural notion of *Woori* (“we”), describes optimal states as recognized relationally rather than individually. Tan et al.’s (2020) study of *ngeli* in Javanese gamelan identifies a state whose fulfillment is encoded in the condition of the ensemble as a whole. These accounts demonstrate that collectively located recognition criteria exist in practice—but they do not resolve the theoretical question of how such criteria function as structural features of the practice itself, nor do they connect to the broader problem of where psychology has tended to locate the criterion of collective optimal experience.

The present study addresses this gap through an analysis of *bisosilin*, the evaluative term used by Bunun singers to recognize the completion of *pasibutbut*, the traditional ritual polyphonic chant performed in ritual contexts associated with millet cultivation, prayer, and collective well-being (Kurosawa, 1973; Mabuchi, 1974). Pasibutbut has been documented within ethnomusicological studies of Bunun polyphonic singing across several decades, from early external recordings and observations (Kurosawa, 1973) and the foundational Taiwanese ethnomusicological record of Indigenous music (Lü, 1977) to subsequent diachronic and contemporary analyses of the practice (Wu, 2009). The chant is characterized by a sustained ascending polyphonic structure in which multiple vocal parts enter sequentially and converge into a layered harmonic field, performed within ritual contexts associated with millet cultivation, prayer, and collective well-being. Building on this established scholarly record, the present study does not revisit pasibutbut primarily as a musical structure or heritage object, but treats it as a phenomenological anchor for examining how collective completion is recognized in practice. *Bisosilin* is not a concept introduced by researchers. Rather, it is a living evaluative term used by practitioners at the moment of performance to determine whether the chant has properly arrived. This case makes the recognition problem empirically visible in a way that existing laboratory- and survey-based studies cannot. For example, field observations consistently show that determination of whether *bisosilin* has occurred does not depend on any individual’s internal feeling but on whether the whole configuration—vocal, bodily, intentional, and spiritual—has reached a recognizable state of completion. Elders and experienced chanters can agree that “we did not reach it today,” even when some individuals privately felt moved. This interpretation is based on consistent patterns observed across interviews, rather than on isolated reports. The criterion is public, relational, and located in the group’s configuration—not in its members’ aggregated ratings.

On the basis of this empirical case, this study advances the following theoretical claim: optimal experience is not first generated and only subsequently recognized; rather, generation and recognition occur as aspects of a single relational configuration that becomes recognizable as having reached completion. This is not a weaker version of existing accounts. It is a direct challenge to the epistemic structure underlying them. When generation and recognition are understood in this way, accounts oriented to the individual phenomenal report describe the aftermath of establishment rather than the moment at which the configuration becomes recognizable as complete. This implies that optimal experience is not an internal state to be measured but a condition that exists only insofar as it is collectively recognizable.

This study pursues two connected objectives. Empirically, it identifies the recurrent markers and enabling conditions through which Bunun chanters recognize *bisosilin* as having occurred, drawing on two-stage qualitative fieldwork with 24 participants. Theoretically, it proposes that this recognition structure instantiates a general condition of collective optimal experience: the identity of generation and recognition as aspects of a single structural event. Two analytical tools are developed in service of this argument: a three-layer body grammar model specifying the necessary structural preconditions for collective optimal experience and a process–uncertainty–alignment model explaining the dynamic conditions under which those preconditions are actualized into a moment of establishment. Together, these tools redefine not what collective optimal experience feels like, but what it means for it to have occurred. Together, these contributions shift the focus from what optimal experience feels like to how it becomes recognizable as having occurred.

## 2 Methods

### 2.1 Research design

This study adopts a phenomenologically informed qualitative design to examine how *bisosilin* is recognized and evaluated within *pasibutbut* practice. The analytic focus is not on cataloging what participants felt during optimal performance, but on identifying the markers and conditions that chanters invoke when explaining how they knew whether a performance had “arrived.” This orientation is consistent with the study’s theoretical concern, i.e., rather than treating optimal experience as an internal state to be measured, the analysis focuses on the publicly articulable criteria through which its occurrence is collectively confirmed. The design is aligned with interpretive qualitative approaches that prioritize participants’ evaluative practices rather than prioritizing predefined psychological constructs.

A clear distinction was maintained between the empirical description and the theoretical interpretation throughout the analysis. The Findings section reports patterns grounded in participants’ accounts, while theoretical implications are developed separately in the Discussion. This boundary was treated as a methodological commitment rather than a presentational convention. That is, the Findings section presents what participants said and how they said it, while the Discussion section performs the theoretical elevation from those patterns to the structural argument.

### 2.2 Participants and sampling

A total of 24 adult Bunun participants with direct *pasibutbut* singing experience participated in the study. Owing to traditional ritual conventions, *pasibutbut* is performed exclusively by men; accordingly, all participants with singing experience were male. Recruitment was conducted through local cultural networks and officially registered intangible cultural heritage preservation groups in Taiwan. At the time of data collection, *pasibutbut* preservation was organized primarily at the group level, with 12 registered preservation groups nationally (Ministry of Culture, Taiwan, field data, 2020). Accordingly, sampling followed group-based structures rather than individual selection.

Data collection proceeded in two stages. The first stage (December 2015, 4 days) included 14 participants representing different roles within *pasibutbut* practice: one elder, one instructor, and twelve experienced performers from a Bunun performing group based in Taipei. This role-based sampling strategy was designed to capture diverse experiential perspectives across different forms of participation, generating a broad initial range of evaluative accounts. The second stage (December 2016, 5 days) involved 10 participants who were assigned the letters A through J. Participants A and B were Bunun elders from Zhuoxi Township, Hualien; the remaining eight were members of the Bunun Cultural Development Association in Xinyi Township, Nantou County—officially designated by the Ministry of Culture (Taiwan) in 2010 as a preservation group for Bunun traditional musical arts. Participants in this stage ranged from approximately 50–60 years of age, with 7–9 years of singing experience and a practice frequency of approximately once per week. All participants provided informed consent verbally prior to participation in accordance with local cultural practices and received modest compensation for their time.

### 2.3 Data collection

The interview questions across both stages were organized around three consistent themes: the conditions under which a performance can be considered truly successful, the factors that enable the chant to reach that state, and the factors that prevent it from doing so. This three-pronged structure—positive, enabling, and obstructing conditions—allowed both enabling and boundary conditions of *bisosilin* to be identified through contrast rather than solely through direct description.

The first stage employed open-ended individual interviews and group discussions, generating approximately 150 min of audio recording, 160 min of video recording, and 57,390 words of transcribed interview material. The second stage used a two-step design. In the first step, open-ended interviews were conducted, allowing participants to describe their chanting experiences without constraints. In the second step, a structured interview and a researcher-developed assisted questionnaire were administered in an interview format. The questionnaire’s objective was to elicit explicit verbal elaboration on the evaluative markers and enabling conditions that had emerged in first-stage open-ended interviews; it was not designed to produce a psychometric measure of bisosilin. The 16 items—11 addressing experiential markers of *bisosilin* and 5 addressing conditions participants considered necessary for its occurrence—were derived from the first-stage interview corpus through the following procedure: recurrent evaluative formulations across first-stage participants were extracted, reformulated as first-person statements (e.g., “I felt a sense of becoming one while singing”), and grouped under the two dimensions above. Items were rated on a five-point Likert scale (1 = strongly disagree, 5 = strongly agree), with verbal elaboration required after each response; the elaboration, not the numerical rating, constituted the primary analytic material. Mean scores reported in the Findings function as descriptive indicators of agreement consensus across participants, not as inferential statistical evidence. These items were not intended as a psychometric instrument but as a structured elicitation tool that preserved participants’ own evaluative logic rather than imposing predetermined psychological categories. All second-stage interviews were audio- and video-recorded, yielding approximately 6 h and 10 min of recorded material. Field notes were written after each session to document contextual observations and nonverbal cues. The interviews followed a phenomenologically informed conversational approach rather than a fixed protocol. Questions invited participants to recount specific occasions on which they recognized that bisosilin had or had not been reached, with the analytic interest oriented to participants’ own evaluative formulations rather than to elicit theory-laden responses. The interviewer maintained a position of attentive engagement consistent with the cultural register of the practice—neither adopting a detached interviewer stance nor steering participants toward predetermined categories—and used Mandarin and locally familiar terms while preserving key Bunun evaluative vocabulary (bisosilin, anitu, and others) in the original language. Probing was used selectively to invite elaboration when participants gestured toward but did not articulate evaluative criteria; the research problem of recognition was not introduced directly as a theoretical construct but approached through participants’ accounts of when chanting performances “arrived” or “did not arrive.” Topics covered across both stages were organized around the three themes already described—conditions of successful performance, enabling factors, and obstructing factors—and were addressed indirectly through narratives of concrete performance occasions rather than through abstract questioning about bisosilin as a category.

### 2.4 Supplementary flow measurement

To situate the *pasibutbut* experience in relation to established psychological constructs, an adapted version of the Flow State Scale (Jackson and Marsh, 1996), reformulated across nine dimensions to reflect the *pasibutbut* context, was administered to ten participants in June 2016. The resulting scores are used in this study exclusively as a supplementary empirical reference for identifying points of convergence and divergence between *bisosilin* and standard flow experience—not as a primary analytic tool, not as a criterion for defining *bisosilin*, and not as a basis for the theoretical claims advanced in the Discussion. The flow scale results function as descriptive contextual comparisons relative to established flow constructs and are not treated as evidence for defining or measuring *bisosilin*.

### 2.5 Analytic strategy

The analytic approach belongs to phenomenologically informed interpretive analysis of field material rather than to inductive coding traditions such as Grounded Theory or to structured content analysis. The procedure does not aim at exhaustive line-by-line coding for the development of theoretical categories from data, nor at thematic frequency mapping. Instead, the analysis foregrounds participants’ own evaluative formulations and traces how these formulations function within the practice of bisosilin recognition. The analytic material includes verbal transcripts as the primary record, supplemented by attention to metadiscursive sonic expressions (gestures of voice, breathing, sung interjections) and to nonverbal cues documented in field notes; these supplementary materials were not coded as a parallel dataset but were drawn upon where they illuminated the evaluative formulations under analysis. The analysis was guided by a specific focus on recognition, not on a description of experience. Rather than coding for general experiential qualities, attention was directed to passages in which participants made evaluative judgments about whether a chanting performance had successfully reached *bisosilin*. Accordingly, the analytic task was therefore not to catalog subjective states but to identify how participants themselves distinguished between performances that had “arrived” and those that had not. This distinction structured the entire analytic process. Specifically, the study does not ask what *bisosilin* feels like, but how participants determined that it had occurred. This analytic focus was consistently applied across all transcripts to ensure comparable coding decisions.

All interview transcripts were produced in Chinese, with key Bunun terms preserved in the original language. The analysis followed an iterative qualitative procedure organized across three stages.

In the first stage, all transcripts were reviewed holistically to identify segments in which participants referred to the success, completion, or failure of a chanting performance. A passage was classified as evaluative when it explicitly or implicitly indicated whether a performance had succeeded, failed, or reached completion, rather than merely describing experiential qualities. This operational distinction was maintained consistently across all transcripts and formed the primary analytic boundary separating data relevant to the recognition problem from a general experiential description.

In the second stage, the identified segments were further differentiated into two types: descriptive accounts of what the experience felt like and evaluative statements indicating recognition of *bisosilin* as having occurred or not having occurred. Only the latter were retained for focused analysis. This differentiation was applied iteratively across multiple readings of each transcript, with decisions reviewed and revised as patterns became clearer across the dataset.

In the third stage, recurrent patterns across participants were compared to identified shared markers and enabling conditions associated with recognition. Only patterns recurring across multiple participants were retained as analytic categories. Single-participant accounts, however vivid, were documented as contextual material but not elevated to the status of shared patterns. Analysis proceeded until no new recognition patterns emerged across participants, indicating thematic saturation. Categories were refined through repeated comparisons across transcripts until stable patterns of recognition emerged.

### 2.6 Analyst position and validity

The analysis was conducted by the lead author, whose engagement with the cultural and performative context of *pasibutbut* extends across multiple fieldwork stages and is sustained by a research orientation directed toward Bunun ritual chant and embodied performance. The lead author’s Bunun background and sustained engagement with pasibutbut informed the cultural and linguistic sensitivity required to attend to participants’ descriptions, while the analysis remained focused on recurring experiential and performative markers reported by participants rather than on personal familiarity alone. This positional configuration is treated as part of the analytical condition of phenomenologically informed interpretive work, rather than as a limitation remediable by external coder agreement alone. The single-analyst design is appropriate to the interpretive-phenomenological lineage of the analysis: the procedure depends on sustained engagement with the cultural register and evaluative vocabulary of the practice, and would not necessarily be strengthened by partitioning the interpretive work across analysts without comparable engagement. To prevent overinterpretation, analytic interpretations were repeatedly checked against participants’ original formulations, with particular attention given to preserving the distinction between participants’ own evaluative language and the study’s subsequent theoretical interpretation. Attention was given to avoiding the projection of theoretical concepts onto participants’ accounts by grounding all interpretations in explicitly articulated evaluative language. Discrepant accounts—cases in which participants’ descriptions did not fit emerging patterns—were documented rather than suppressed to preserve the full range of evaluative practices represented in the data.

The identification of recognition markers is based on an interpretive analysis of interview data rather than on direct behavioral measurement. While this approach allows access to participants’ evaluative practices as they articulate them, it does not capture real-time processes of recognition during performance. The distinction between what participants report about recognition and what recognition involves as a live perceptual event is acknowledged as a boundary condition of the present study. Future research may complement this approach with real-time observational or physiological measures to capture recognition as it unfolds during performance.

## 3 Findings

The following descriptions remain grounded in participants’ accounts and are presented in analytic terms only to clarify recurring patterns, not to impose a theoretical structure. What emerges consistently across the data is not a set of subjective feelings but a set of recurring signs through which participants determined whether *bisosilin* had occurred. The participants did not consult their individual internal states to make this determination. Rather, they attended to whether the performance as a whole had reached a state they could describe, point to, and confirm with others. The findings are organized into three parts: the experiential markers participants relied on to recognize *bisosilin*; the conditions they considered necessary for its occurrence; and an empirical contrast with standard flow experience. These patterns were identified through repeated comparisons across participants’ accounts rather than through isolated instances.

### 3.1 Experiential markers: how participants recognized that bisosilin had been reached

#### 3.1.1 Unity as an indication that the performance had come together

Across both stages of fieldwork, participants repeatedly referred to a sense of unity as one of the clearest signs that *bisosilin* had been reached. The questionnaire responses revealed strong agreement with the item “experiencing a sense of becoming one while singing” (mean = 4.6), but qualitative accounts clearly revealed that this unity was not described as a merely private feeling. Rather, chanters relied on it as an indication that voices, bodily effort, and shared orientation had converged in a way that was recognizable to all. Participant I described fulfillment as occurring when “voice and power are all merged together” (I-2), with resonance proceeding “without any divergence” (I-3). Participant B described a state in which “there is no distinction in the heart” (B-4) and noted that in that moment, it no longer felt as though “these [individual] people” were harmonizing (B-1)—a formulation suggesting that the performance had been taken as a sign that something beyond individual effort had come into being. Participant B further described connections reaching toward ancestral presences, “feeling like the ancestors (*anitu*)” (B-2), thereby situating the recognition of unity within an intergenerational field of meaning. Participants G and I summarized this state with formulations such as “being joined together” (G-4) and “oneness” (I-1). Participant F noted that “when singing well, everything feels right” (F-1), indicating that unity was understood as extending beyond the singers themselves to encompass the performance environment as a whole. Taken together, these accounts suggest that participants did not treat unity as an optional emotional accompaniment to a performance, but as one of the primary signs by which they determined that the chant had properly arrived.

#### 3.1.2 Absorbed attention as a sign of sustained alignment

The participants described a state of concentrated attention during *bisosilin,* with a specificity pointing beyond mere subjective focus (Q6, Q7; mean = 4.8). Participant A described being “very lucid in consciousness” (A-2), a clarity that caused external distractions to vanish entirely. Participant F described this as a form of “mental concentration” (F-3) directed toward achieving “collective solidarity” (F-4). Participant J noted that during *bisosilin*, one “does not feel tired but feels very energized” (J-1) and “very comfortable” (J-2), whereas participant K added that in such moments, one “did not think about being tired” (K-1). A particularly important observation came from Participant J, who noted that if someone’s voice “strays” or “fails to harmonize” (J-4), it immediately triggers a sense of “discomfort or abnormality” (J-5). This observation indicates that the absorbed attention associated with *bisosilin* was not passive immersion but active monitoring. Participants relied on the quality of their attention—and specifically on the immediate detection of deviation—as an ongoing sign of whether the performance was still maintaining the state of alignment associated with *bisosilin*. The presence of deviation-triggered discomfort functioned as a real-time indicator that the performance had departed from the recognized state.

#### 3.1.3 Harmony as a sign that the performance had stabilized

Harmony was consistently described not only as a pleasant affective state but also as a sign that the performance had reached a stable state as a whole (Q8, Q9; mean = 4.6). Participant G directly named this “a feeling of harmony” (G-1), while participant E linked it to somatic comfort: “very comfortable” (E-1). Participant F connected harmony explicitly to the ritual’s cosmological purpose, noting that when *bisosilin* occurred, “the millet harvest [and all things] would be well” (F-2), indicating that harmonic stability was taken as a sign that extended beyond the sonic domain into the ritual and cosmological dimensions of the performance. Participant D added a sense of “gratitude toward the elders” (D-1), showing that participants’ recognition of harmony incorporated awareness of the traditional social order as part of what “being in the right state” meant. Across these accounts, harmony was relied upon not as a subjective feeling of pleasantness but as a perceivable indication that the performance had settled into the appropriate state.

#### 3.1.4 Mastery as a sign of individual integration into the collective

Technical mastery was described in terms that clearly distinguished it from personal skill display (Q10, Q11; mean = 3.9). Participant B described a transparency in which “the entire voice comes out” (B-3). Participant C characterized the state as being “high-pitched” (C-1), “stable” (C-2), and marked by increasing ease in “opening one’s mouth” (C-3). Participant K described a specific “state” (K-3) of being “very clear” (K-4), in which breath control and part-transitions occurred naturally without conscious deliberation. Participants consistently described mastery not as a demonstration of individual capability but as the condition of having been properly absorbed into the group’s functioning. Technical fluency was taken as a sign that one’s individual contribution had been integrated into the whole—a condition that participants recognized as necessary for *bisosilin* to be recognized, but not identical to it. A singer might feel technically in control while the collective state had not yet arrived, and participants were clear about this distinction.

#### 3.1.5 Auditory expansion as a sign that a shared acoustic field had formed

Distinctive auditory experiences were consistently associated with *bisosilin*, and participants relied on them as indications that the sonic field of the performance had reached a particular state (mean = 3.2). Participant A described hearing “completely harmonized sounds” (A-3) and a spatial sensation “like bees flying, surrounding us” (A-4). Participant E described a “humming” resonance in the ears (E-2). Participant B characterized the sonic experience as a “3D space” (B-7), while another account included the experience of “not hearing others’ voices or people who have passed away coming back” (A-5)—a description in which the sonic field was taken as a sign that the performance had transcended ordinary individual vocal sources. These accounts are not presented merely as unusual personal sensations but as descriptions that participants used to distinguish performances that had reached *bisosilin* from those that had not. They describe specific auditory qualities that participants recognized as marking the difference between a performance that had arrived at *bisosilin* and one that had not. The spatial, enveloping, and unified quality of the sound was relied upon as one of the perceivable signs that the shared acoustic state had been reached.

#### 3.1.6 Cultural belonging as normative confirmation

Cultural and ancestral belonging functioned as a normative frame through which participants confirmed that *bisosilin* had been properly recognized (mean = 3.6). Participant A stated that when the singing “reaches the peak,” one “gets goosebumps and truly feels like a Bunun” (A-1)—a formulation connecting a physiological response to cultural identity at the moment of recognition, suggesting that the experience was taken as a sign of having properly enacted Bunun tradition. Participant B described the sound as a realization that “this is what the ancestors’ voices sounded like” (B-6), situating recognition within an intergenerational continuity that extended beyond the present singers. Participants D and G reported goosebumps as a physiological accompaniment to recognition (D-2; G-3), indicating that a bodily response was taken as a confirming signal that the performance had met the normative standard. These accounts suggest that, for participants, the recognition of *bisosilin* was not culturally neutral. Rather, it was evaluated against an ancestral standard, and cultural belonging was itself part of what made the recognition complete.

### 3.2 Enabling conditions: what participants considered necessary for *bisosilin* to occur

In addition to experiential markers, participants consistently referred to a set of conditions they considered necessary for *bisosilin* to occur. These conditions do not define *bisosilin* itself but describe the enabling context within which recognition becomes possible. Participants clearly distinguished between having these conditions in place and actually reaching *bisosilin* as the conditions could be present without the state being achieved, but without them conditions, participants agreed that *bisosilin* could not be reached.

Shared upward intentionality received the highest agreement among the enabling conditions (mean = 4.8). Participant K identified “having an upward heart” (K-1) as essential to the possibility of *bisosilin*, and Participants I and B both emphasized that “everyone’s heart and *qi* must go upward” (I-2; B-4). This condition was described as a collective rather than an individual requirement. In other words, it was not sufficient for one or two singers to hold this orientation—all participants needed to be directed toward the same upward movement. The optimal body–mind state was equally emphasized (mean = 4.7). Participant F stressed “personal character” (F-3) as a precondition, while Participant A described the necessity of “reverence toward the Millet Deity or ancestors” (A-2), indicating that moral and spiritual preparation were understood as prerequisites rather than accompaniments to the chant. Similarly, fundamental skill mastery was considered necessary (mean = 4.7): Participant C specified that insufficient technique prevents the breath from sustaining the required sound structure (C-2), pointing to technical competence as a floor condition below which *bisosilin* cannot be reached regardless of other factors. Collective tacit understanding (mean = 4.6) was described by Participant B as “feeling the other without speaking” (B-5) and Participant J described it as a “long-term practice and shared life experience” (J-2)—an implicit coordination that participants regarded as built over time rather than achievable through explicit communication. Finally, sequential progression (mean = 4.5) was consistently emphasized across participants. For example, Participant E noted the necessity of “the accumulation of breath” (E-1); Participant D described a “rising temperature” (D-3); and Participant A referred to “reaching the peak” (A-1) as a culmination requiring a gradual approach rather than direct attainment.

These conditions, in and of themselves, do not determine that *bisosilin* has been achieved. Rather, they define the enabling context within which recognition becomes possible—a distinction that has direct implications for the theoretical account developed in the Discussion. Participants’ accounts consistently distinguished between the presence of these conditions and the actual recognition of *bisosilin* as having occurred.

### 3.3 Empirical contrast with standard flow experience

The adapted flow state scale produced a mean overall score of 4.09 across the nine flow dimensions, indicating that *pasibutbut* singing is associated with a high degree of flow-related experiential characteristics. Two dimensions, however, produced patterns that diverge from standard individual flow experience in ways that participants themselves described and that are relevant for situating *bisosilin* within the broader literature.

The dimension of time transformation received the lowest mean score (3.4). Participants did not describe losing track of time during *bisosilin*; rather, they maintained acute awareness of temporal sequences—breath timing, part-entry sequences, and the moment of collective ascent—as structural requirements for sustaining the performance. Participant accounts consistently reflected this temporal awareness as a feature of skilled participation rather than an indication of insufficient absorption. Losing track of time, in the context of *pasibutbut*, would undermine the collective coordination that participants identified as necessary for *bisosilin* to be recognized.

Rather than the loss of self-consciousness associated with individual flow, participants described an expansion of self that incorporated ancestral presence. Participant B’s account—that the performance no longer felt like “these people” harmonizing but like ancestral voices converging (B-1; B-6)—and Participant A’s description of “people who have passed away coming back” (A-5); both describe a widening rather than a dissolution of experiential scope. Self-awareness did not disappear but rather, extended outward to encompass a shared experiential field that included presences beyond the immediate group. These empirical contrasts are noted herein as data-level observations rather than theoretical claims. Their implications for how *bisosilin* is situated relative to flow theory are addressed in the Discussion. These observations are reported herein solely to situate *bisosilin* empirically, not to evaluate or revise flow theory.

## 4 Discussion

The findings reveal a consistent evaluative logic across participants, contexts, and performance occasions. Specifically, the recognition of *bisosilin* did not depend on how intensely any single individual felt, but on whether the performance as a whole had reached a state that participants could perceive, describe, and confirm with others. This pattern can be understood as an empirical instance of the recognition problem identified in the Introduction. The following discussion builds on this empirical base to develop a theoretical account of collective optimal experience as a structural event—proceeding from the relocation of the recognition criterion to the necessary conditions, the dynamic mechanism, and finally to the identity of generation and recognition.

### 4.1 Relocating the criterion: shifting the unit of analysis

The findings present a case that cannot be adequately described within existing collective optimal experience frameworks—not because the case is culturally exotic but because the evaluative logic it reveals is structurally different from that assumed by the dominant research tradition. In most studies of group flow, team flow, and collective effervescence, the occurrence of a collective state is confirmed through individual self-reports: participants rate their own experiences, and the collective is inferred from the distribution of those ratings (Peifer et al., 2024; Pels et al., 2018). Even questionnaire items phrased in “we” terms preserve the individual as the unit of judgment. However, the decisive question remains: how strongly did *you* feel it?

The *bisosilin* data suggest a different logic. The findings indicate that elders and experienced chanters can agree that “we did not reach it today,” even when some individuals privately felt moved—and conversely, can recognize a complete performance even when a particular singer felt tired or distracted. The criteria of fulfillment were described as public and relational rather than private and statistical. The state of the shared field—the sonic, bodily, and intentional configuration available to joint perception—was what participants attended to rather than the aggregation of personal reports. This reverses the standard direction of inference. Thus, rather than deriving the collective from individual ratings, participants took the quality of the relational configuration as primary and treated individual feelings as reflections of how well that configuration had come into being.

The question is, therefore, no longer “what did participants experience?” but “Under what conditions does a practice become recognizable as complete?” This reframing is consistent with participatory and interaction-oriented accounts of intersubjectivity that treat the interaction process itself as the primary site of meaning (De Jaegher and Di Paolo, 2007). Unlike many general embodiments, the concept of body grammar developed in this study is concerned less with how the body enables cognition than with how collective configurations become recognizable as complete. The task of the following sections is to specify the structural conditions under which such a relational configuration becomes possible and, crucially, how it crosses the threshold from occurring to being established.

### 4.2 Body grammar as the enabling structure

To account for the structural conditions identified, this study proposes the concept of body grammar—a three-layer model that specifies the experiential preconditions for collective optimal experience. The term grammar designates an analytical structure rather than a purely metaphorical description, i.e., a set of rule-governed, collectively internalized action and perception structures that organize coordinated practice and provide the framework within which completion can be recognized. The three layers proposed below are not presented here as inductively derived empirical categories but as theoretical abstractions from recurring experiential and performative markers reported across the pasibutbut fieldwork. The Findings section documented these recurring markers at the level of participants’ own evaluative formulations; the analytical move performed here is to specify the structural conditions those formulations imply. Specifically, action grammar abstracts the recurring bodily-coordination markers documented at §3.1.4 (mastery as a sign of individual integration into the collective) and §3.2 (sequential progression as an enabling condition); perceptual grammar abstracts the recurring monitoring-and-adjustment markers documented at §3.1.2 (absorbed attention with deviation detection) and §3.1.5 (auditory expansion as the formation of a shared acoustic field); and consciousness grammar abstracts the recurring normative-orientation markers documented at §3.1.6 (cultural belonging as normative confirmation) and §3.2 (shared upward intentionality; optimal body–mind state as enabling condition). The proposal is therefore not that the data prove a three-layer structure, but that a three-layer structure provides the most parsimonious analytical articulation of the conditions implicit in the recurring markers participants reported. Three interrelated layers are identified, each corresponding to a dimension of the recognition markers described in the Findings section.

Action grammar refers to the stable, rule-governed system of bodily actions and spatial arrangements that organize collective practice: circle formation, coordinated breathing, rhythmic body movement, and vocal entry sequences structuring *pasibutbut*. The findings indicate that participants regarded these as physical prerequisites. For example, Participant C specified that insufficient technique prevents the breath from sustaining the required sound structure (C-2). By establishing shared timing and spatial configuration, action grammar enables participants to form what cognitive science terms a feed-forward model: a shared anticipatory structure that allows each participant to orient toward others’ actions before completion (Vesper et al., 2010). In the present analysis, action grammar is proposed as the physical foundation upon which subsequent layers of coordination depend.

Perceptual grammar refers to the shared bodily perceptual standards through which participants continuously monitor and adjust the collective state. These findings indicate that this occurred in *pasibutbut* through the listen–catch mechanism: participants detected others’ sonic positions and immediately adjusted their own vocal output to fit within the emerging structure. Participant J’s account of deviation-triggered discomfort (J-4; J-5) illustrates this monitoring function directly. De Jaegher and Di Paolo's (2007) account of participatory sense-making provides a useful interaction-based framework for understanding how coordination can acquire relative autonomy from individual intention. Subsequent autopoietic-enactivist work has extended this framework to the formation of shared sensorimotor and interactional structures across participants in sustained collective practice (Di Paolo et al., 2018), establishing a broader theoretical context within which practice-specific coordination structures can be analysed. In the present analysis, perceptual grammar is proposed as the practice-specific mechanism through which such coordination is continuously renewed in *pasibutbut*—a distinction that the participatory sense-making account describes in general terms but does not specify at the level of particular bodily operations.

Consciousness grammar refers to the shared normative framework and intentional orientation governing the nature and direction of practice. The findings indicate that *pasibutbut* was framed by participants as an offering to ancestral spirits rather than as a performance or competition—a framing described by Participant F as connected to the ritual purpose of ensuring the harvest (F-2) and by Participant A as requiring reverence toward the Millet Deity (A-2). Searle's (1995) account of collective intentionality and Rappaport's (1999) analysis of ritual’s normative efficacy provide compatible frameworks for understanding how shared intentional orientation can transform the evaluative weight of action. In the present analysis, consciousness grammar is proposed as the condition through which the same bodily actions may carry different recognition implications, depending on the normative frame within which they are performed.

**Figure 1:**
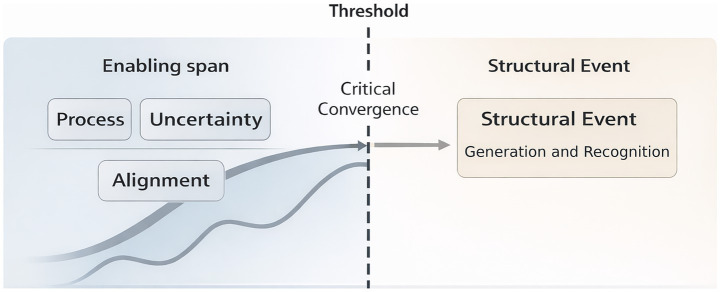
Collective optimal experience emerges when body grammar operates under PUA conditions and converges at a critical threshold. The left zone represents the enabling condition span during which body grammar is dynamically activated under PUA conditions. The vertical line indicates the threshold of critical convergence. The right zone designates the structural event in which generation and recognition are constituted as structurally inseparable aspects of establishment. Body grammar enables but does not establish; establishment occurs only at the moment of critical convergence.

The dynamic relationships among the PUA conditions, body grammar coupling, critical convergence, and the structural event of establishment are presented in Figure 1.

Table 1 summarizes the three layers and their functional roles.

**Table 1:** Three-layer body grammar model and its function in collective optimal experience.

Grammar layer	Definition	Proposed function in COE	Structural limitation
Action grammar	Rule-governed bodily action and spatial arrangement: circle formation, coordinated breathing, vocal entry sequences	Generates collective physical foundation and shared anticipatory timing	Necessary but not sufficient: stable action does not guarantee perceptual alignment
Perceptual grammar	Shared bodily perceptual standard: listen–catch mechanism, real-time sonic monitoring and mutual adjustment	Provides detection and correction mechanism for ongoing collective alignment	Necessary but not sufficient: perceptual coordination does not guarantee intentional convergence
Consciousness grammar	Shared normative and intentional framework: ritual framing, directedness toward ancestral offering, normative constraint	Frames the nature and direction of practice; defines what counts as completion	Necessary but not sufficient: correct framing does not guarantee that the structural threshold is crossed

COE = collective optimal experience. Three layers are proposed as necessary structural preconditions of the COE. They do not, in and of themselves, however, determine its establishment.

The critical implication follows directly from the data and from the logic of the model: body grammar enables but does not establish. All three layers constitute necessary conditions—the findings indicate that without any one of them, participants did not consider *bisosilin* achievable. However, their simultaneous presence was not described as sufficient. Participants consistently distinguished between conditions being in place and the state actually being reached—a distinction directly expressed in accounts where all conditions appeared satisfied yet *bisosilin* was still not recognized as having occurred. Whereas the grammar defines the possibility space, the argument advanced herein is that it does not determine which possibilities are actualized. This distinction between structural conditions and structural events is the theoretical hinge upon which the next section turns.

### 4.3 From structural conditions to structural event: the process–uncertainty–alignment (PUA) model

If body grammar constitutes the necessary conditions for collective optimal experience, the remaining question is as follows: under what dynamic conditions do those structural conditions cross the threshold from enabling to actualizing? The process–uncertainty–alignment (PUA) model proposed here specifies the interaction conditions through which body grammar is dynamically activated—and through which the system may cross the threshold into what this study terms critical convergence.

The three PUA conditions are proposed as co-constitutive dimensions of the interaction field rather than sequential variables. The PUA model is not intended as an exhaustive taxonomy of interactional dimensions, but as a minimal analytical framework sufficient to account for the transition from coordination to establishment observed in this case. Process refers to the temporal continuity of group interaction, i.e., the sustained engagement allowing participants to gradually attune themselves to one another’s rhythms, breathing, and sonic positions. The findings indicate that participants consistently described *bisosilin* as requiring a gradual approach. Participant A referred to “reaching the peak” as a culmination (A-1), while Participant E emphasized “the accumulation of breath” (E-1). Without temporal continuity, the iterative adjustments necessary for alignment cannot accumulate. Uncertainty refers to the irreducible openness of the interaction, i.e., the microvariations in breath timing, vocal quality, and bodily response that cannot be fully predicted. The listen–catch mechanism described by participants—an active, ongoing calibration rather than the reproduction of a fixed template—implies that productive engagement with uncertainty is a feature rather than a defect of the practice. Without it, the practice would degenerate into mechanical repetition, and perceptual grammar would lose its active calibration function. Alignment refers to the dynamic, progressive convergence of action and perception across participants—not a static endpoint but an ongoing relational process through which individual contributions become increasingly integrated into a sustainable collective structure.

Existing coordination models are primarily concerned with how alignment emerges from local interaction dynamics (Riley et al., 2011; Tognoli et al., 2020). In contrast, the PUA model proposed herein focuses on the condition under which such alignment becomes recognizable as an establishment. This distinction—between alignment occurring and alignment being established—is the theoretical gap that the PUA model is designed to address. Within this framework, the three subsections that follow address successive aspects of the same dynamic system rather than three condition-specific mechanisms: §4.3.1 specifies how body grammar undergoes progressive coupling as Process, Uncertainty, and Alignment jointly operate; §4.3.2 specifies the threshold-crossing at which this coupled system reaches a qualitatively distinct state of integration; and §4.3.3 specifies the structural relationship between generation and recognition that holds at that threshold. The three PUA conditions are co-constitutive throughout—they do not map onto the three subsections one-to-one but jointly condition each of them.

#### 4.3.1 Dynamic coupling

Within the PUA field, the argument advanced is that body grammar across participants undergoes dynamic coupling: the three layers become increasingly coordinated as the performance progresses, not through mechanical synchronization but through the progressive integration of multiple degrees of freedom into a sustainable relational structure. The findings support this characterization, and practitioners described the process with consistent precision. “Catching the sound” was described not as pitch imitation but as real-time adjustment of timing, breath, and tonal position. “Locking” referred to a state in which the individual voice had formed a stable connection with the whole. “Pulling back” marked the active correction of deviation before it propagated through the system. These descriptions are consistent with the error-correction and phase relocking mechanisms documented in joint action research (Repp and Keller, 2008; Vesper et al., 2010), although the present analysis is concerned with a distinct question, i.e., not how coordination is maintained but how it comes to be recognized as established.

#### 4.3.2 Critical convergence

The field data consistently reveal a qualitative discontinuity that linear accumulation models cannot capture. The findings indicate that participants did not describe *bisosilin* as the upper end of a continuum of quality but as a moment in which “everything suddenly comes together”—a qualitative change in the state of the whole. The expression recurring across participants—“it flows well but has not yet arrived”—is theoretically decisive: fluency, in participants’ accounts, was not fulfilled. Fluency denotes dynamic stability within the PUA field, whereas fulfillment denotes the crossing of a threshold into a qualitatively different state.

In the present study, the term critical convergence is used to name the moment when alignment becomes an established fact—a nonlinear transition in which the interaction system shifts from distributed local adjustment to integrated global stability. Research on coordination dynamics has described phase transitions and self-organizing stable states in social interaction (Tognoli et al., 2020; Riley et al., 2011), and these accounts provide a useful structural analogy. The argument advanced herein is that critical convergence is a specific instance of such a transition: the moment at which body grammar, activated under PUA conditions, crosses the threshold into the established state of *bisosilin*.

#### 4.3.3 The Identity of generation and recognition

Critical convergence has a consequence that is both empirically grounded in the findings and theoretically significant. In standard psychological accounts, generation and recognition are treated as temporally separated phases: an experience occurs, and it is subsequently recognized as having occurred. This separation underlies the self-report methodology in which participants are asked, after the fact, how strongly they felt something. The argument advanced here is that, under the specific conditions of coordinated collective practice examined in this study, this separation does not hold in the standard form. Identity here is structural rather than merely temporal: the relational configuration that brings the practice into completion is the same configuration through which that completion becomes recognizable to participants. Accordingly, accounts oriented to the individual phenomenal report describe the aftermath of establishment rather than the moment at which the configuration becomes recognizable as complete. This methodological implication follows directly from the data pattern described below.

The findings indicate that participants consistently recognized *bisosilin* at the moment of its occurrence, not upon reflection. When the sonic field “locked,” the recognition of completion was described as simultaneous with the locking itself—there was no reported interval between the event and its confirmation. Although recognition appeared to be internal to the event, the negative direction of the evidence is equally informative. That is, when a voice part persistently deviated and the system failed to converge, chanters consistently reported that “the catching was not smooth”—a shared assessment of whether the structural threshold had been crossed, not a statistical aggregation of individual dissatisfactions.

On the basis of these field patterns, this study advances the following theoretical claim.

In the high-quality collective practice of the kind examined here, the claim is not that generation and recognition are phenomenally indistinguishable processes. Rather, under the specific conditions of coordinated collective practice examined in this study, the relational configuration that brings the practice into completion is the same configuration through which that completion becomes recognizable to participants. This is the sense in which generation and recognition can be understood as two aspects of a single structural event rather than two temporally separated phases. This formulation should not be interpreted as a universal claim across all forms of collective experience, but as a structural possibility that becomes observable under specific conditions of highly coordinated collective practice. When body grammar reaches critical convergence under process–uncertainty–alignment conditions, the moment at which the configuration becomes complete is the moment at which it becomes recognizable as such, not because two distinct processes happen to coincide in time but because the configuration’s completion and its recognisability are aspects of the same structural achievement.

The implication, within this form of collective practice, is that experience is not first generated and then recognized but becomes identifiable only insofar as it is recognizable within the relational configuration. This does not deny the reality of individual feelings, but instead, it proposes that individual feelings, in this context, function as reflections of the relational configuration rather than as its ground. The account developed here proposes that, at the moment when body grammar reaches critical convergence under PUA conditions, the experience is both generated and identifiable as having occurred.

### 4.4 Theoretical implications and research directions

The argument advanced in this study has implications that extend beyond the specific case of *bisosilin*, although the scope of those implications requires careful qualification. At the conceptual level, the study identifies and names the recognition problem, specifically, the questions of by what criterion, the location where *bisosilin* may have occurred, and whether a collective state is confirmed as having occurred, as a structural gap in the literature on collective optimal experience. The answer proposed herein does not apply universally, but rather, it establishes that the individualist assumption embedded in existing frameworks is not necessary and provides an empirically grounded alternative. If the criterion of collective optimal experience can be located in the relational configuration rather than in individual states—as the *bisosilin* findings suggest—then existing research frameworks have been measuring the aftermath of an event whose occurrence they have not directly addressed. This suggests a substantial reconsideration of current methodological assumptions, and it reopens a foundational question about what collective optimal experience is, where it resides, and how it can be known to have occurred.

At the methodological level, the identity of generation and recognition—if it holds in other collective practices—has direct implications for research design. Retrospective self-reports, which capture participants’ reflections after the event, may be structurally unable to access the moment of establishment as it occurs. If recognition is internal to the event of completion rather than subsequent to it, then the methodological instruments best suited to capturing collective optimal experience are not *post hoc* questionnaires but real-time observational methods capable of tracking the interaction process as it unfolds. Future research may operationalize recognition by tracking moments of convergence, deviation correction, and collective acknowledgment in real-time interactions—for example, through continuous audio-visual recording analyzed for phase alignment in vocal timing or through coordinated physiological measurement during high-coordination collective tasks. Such approaches complement the present interpretive analysis with behavioral and physiological indices of the structural transitions that participants describe as critical convergence.

At the applied level, the PUA model provides a basis for rethinking what constitutes high-quality collective functioning in professional and organizational contexts. Research on team flow has established that shared goals, communication, and mutual trust are important enabling conditions (Van den Hout and Davis, 2023; Aust et al., 2023). Accordingly, the present analysis complements this by specifying that the decisive consideration is not whether enabling conditions are in place but whether the interaction system crosses the threshold of critical convergence, that is, whether the group reaches a state that can be collectively recognized as complete. This shifts the focus from designing for individual engagement toward designing for the relational conditions under which convergence becomes possible. Contexts in which this distinction may be practically consequential include collaborative musical performances, surgical team coordination, improvisational ensemble work, and any domain in which the quality of the collective outcome is not reducible to the sum of the individual contributions.

The generalizability of these implications is bounded by the case from which they are derived. Whether analogous recognition criteria operate in other cultural or professional practices—whether the identity of generation and recognition characterizes collective optimal states in nonritualized, nonmusical, and nonindigenous contexts—is a question that this study introduces rather than resolves. Although the *bisosilin* case demonstrates that such a structure can exist in practice and can be empirically examined, it does not establish that it is universal.

### 4.5 Limitations

Three limitations require explicit acknowledgment, each arising directly from the design choices described in the Methods section.

First, cultural and case specificity: The analysis is developed through a single cultural case, whereas the body grammar framework has not been operationalized or validated across different cultural traditions, performance practices, or organizational contexts. The *bisosilin* findings are grounded in a specific ritualistic, linguistic, and cosmological context that shapes both the practice and the evaluative vocabulary participants used. Whether structurally analogous recognition criteria exist in other practices and whether the PUA model applies across different forms of collective coordination require comparative empirical examination. The present study establishes a proof of concept, i.e., it does not establish a generalization.

Second, the interpretive nature of recognition coding: The identification of recognition markers is based on an interpretive analysis of interview data rather than direct behavioral measurement. The analytic boundary between evaluative and descriptive passages—while applied consistently and operationalized explicitly—remains a judgment-based distinction rather than one derived from a standardized coding instrument. Different analysts applying the same decision rule might code individual passages differently, particularly in cases where participants’ language moves fluidly between description and evaluation. Although the findings therefore reflect interpretive patterns that are grounded in consistent cross-participant accounts, they cannot claim the replicability of behavioral coding with established interrater reliability.

Third, the absence of real-time measurement: This study relies on retrospective interview accounts of recognition rather than on observations or measurements of recognition as it unfolds during performance. The distinction between what participants report about how they recognized *bisosilin* and what recognition involves as a live perceptual and interactional event is acknowledged as a boundary condition of the present study. Retrospective accounts are subject to *post hoc* rationalization, memory reconstruction, and the influence of culturally available evaluative vocabularies. The convergence of accounts across participants and contexts strengthens the credibility of the patterns identified, but it does not substitute for real-time evidence of the recognition process. Future research integrating continuous observational or physiological methods with the interpretive framework developed herein would provide a more complete account of how critical convergence is produced and recognized in practice.

## 5 Conclusion

Existing research explains how collective experience emerges but not how it is recognized as having occurred. The present study addresses this recognition problem by demonstrating, through two-stage qualitative fieldwork with 24 Bunun *pasibutbut* chanters, that the criterion of collective optimal experience does not reside within individual consciousness. Hence, the findings indicate that the recognition of *bisosilin* depends not on how any individual participant feels, but on whether a specific relational configuration—organized through three layers of body grammar and actualized under process–uncertainty–alignment interaction conditions—has crossed the threshold of critical convergence.

At that threshold, the argument advanced herein is that generation and recognition do not follow one another but are constituted as structurally inseparable aspects of establishment. Accordingly, the criterion of collective optimal experience is located in the relational configuration rather than in individual states. Within this form of collective practice, optimal experience becomes identifiable only insofar as it is recognizable within the shared configuration—not as an internal state that individuals possess and subsequently report. Rather, it is a condition that is established through the completion of the relational structure itself.

The recognition problem cannot be resolved by better measurement instruments alone as it also reflects a deeper assumption about where the authority of recognition is presumed to reside. The *bisosilin* case suggests that this authority lies in the configuration of the group rather than in its members’ ratings—and that this relocation is not a theoretical revision but an empirical reality for the practitioners examined here. Whether analogous criteria operate in other collective practices is a question that this study opens rather than forecloses.

This study does not introduce a new type of collective peak experience but rather redefines the generative mechanism and establishment condition of collective optimal experience as a structural event.

## Data Availability

The datasets presented in this article are not readily available because the qualitative material underlying this manuscript is not publicly available and cannot be shared upon request because it includes context-sensitive interviews and field materials that could compromise participants’ privacy and cultural confidentiality. The article presents a theoretical framework informed by these materials rather than a shareable dataset. Requests to access the datasets should be directed to langui@gmail.com.
